# 2025 Jack Kenney Award for Outstanding Service

**DOI:** 10.1128/jb.00553-25

**Published:** 2026-01-22

**Authors:** George A. O’Toole

**Affiliations:** 1 Department of Microbiology and Immunology, Geisel School of Medicine at Dartmouth 12285, Hanover, New Hampshire, USA

**Keywords:** Kenney Award, reviewer, acknowledgement

## EDITORIAL

Since 2009, we have conferred the Jack Kenney Award for Outstanding Service to a reviewer who does extraordinary service for the journal. I had the opportunity to work with Jack in my early days as an editor, including on my first special meeting collection that he helped me put together. Jack was an outstanding production editor who retired in 2010; he died shortly after his retirement.

The recipient of the 2025 Kenney Award is Souvik Bhattacharyya (pictured here with his award).


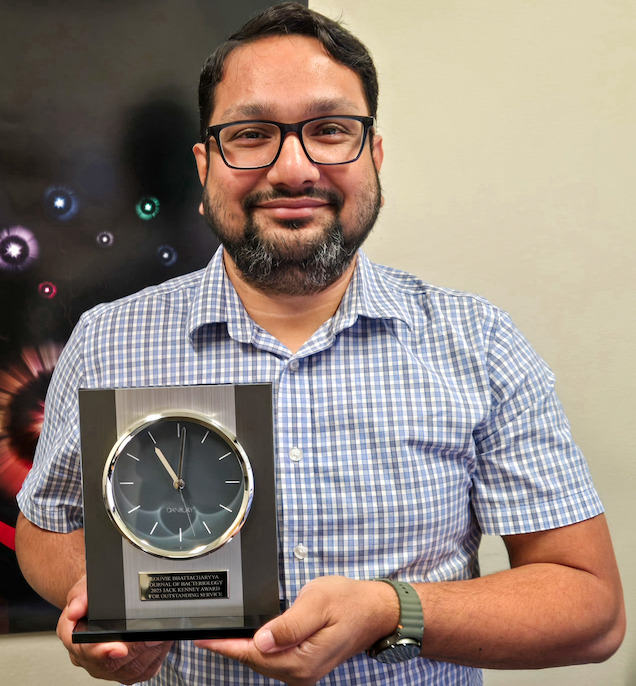


Dr. Bhattacharyya is an Assistant Professor of Microbiology and Molecular Genetics at the University of Texas Health Science Center at Houston. The Bhattacharyya lab studies antimicrobial resistance (AMR) using a wide array of approaches, including experimental evolution, molecular genetics, and computation. His lab seeks to understand vulnerabilities associated with AMR, as well as the contribution of bacterial memory, “necrosignaling,” and global warming to this clinically relevant process.

Over the past year, Dr. Bhattacharyya reviewed 10 manuscripts for JB without declining a single review. This is a fantastic contribution to the journal, and I could not be more pleased to have Souvik be this year’s Kenney Award recipient.

The editors are pleased to recognize Dr. Bhattacharyya’s outstanding service to JB, to ASM, and to society publishing.

